# Automatic Analysis of Cellularity in Glioblastoma and Correlation with ADC Using Trajectory Analysis and Automatic Nuclei Counting

**DOI:** 10.1371/journal.pone.0160250

**Published:** 2016-07-28

**Authors:** Oliver Eidel, Jan-Oliver Neumann, Sina Burth, Pascal J. Kieslich, Christine Jungk, Felix Sahm, Philipp Kickingereder, Karl Kiening, Andreas Unterberg, Wolfgang Wick, Heinz-Peter Schlemmer, Martin Bendszus, Alexander Radbruch

**Affiliations:** 1 Department of Neuroradiology, University of Heidelberg Medical Center, Heidelberg, Germany; 2 Department of Neurosurgery, Division Stereotactic Neurosurgery, University of Heidelberg Medical Center, Heidelberg, Germany; 3 Department of Psychology, School of Social Sciences, University of Mannheim, Mannheim, Germany; 4 Department of Neuropathology, University of Heidelberg Medical Center, Heidelberg, Germany; 5 Neurology Clinic, University of Heidelberg Medical Center, Heidelberg, Germany; 6 Department of Radiology, German Cancer Research Center (DKFZ), Heidelberg, Germany; 7 German Cancer Consortium (DKTK), Heidelberg, Germany; University of Pécs Medical School, HUNGARY

## Abstract

**Objective:**

Several studies have analyzed a correlation between the apparent diffusion coefficient (ADC) derived from diffusion-weighted MRI and the tumor cellularity of corresponding histopathological specimens in brain tumors with inconclusive findings. Here, we compared a large dataset of ADC and cellularity values of stereotactic biopsies of glioblastoma patients using a new postprocessing approach including trajectory analysis and automatic nuclei counting.

**Materials and Methods:**

Thirty-seven patients with newly diagnosed glioblastomas were enrolled in this study. ADC maps were acquired preoperatively at 3T and coregistered to the intraoperative MRI that contained the coordinates of the biopsy trajectory. 561 biopsy specimens were obtained; corresponding cellularity was calculated by semi-automatic nuclei counting and correlated to the respective preoperative ADC values along the stereotactic biopsy trajectory which included areas of T1-contrast-enhancement and necrosis.

**Results:**

There was a weak to moderate inverse correlation between ADC and cellularity in glioblastomas that varied depending on the approach towards statistical analysis: for mean values per patient, Spearman’s ρ = -0.48 (p = 0.002), for all trajectory values in one joint analysis Spearman’s ρ = -0.32 (p < 0.001). The inverse correlation was additionally verified by a linear mixed model.

**Conclusions:**

Our data confirms a previously reported inverse correlation between ADC and tumor cellularity. However, the correlation in the current article is weaker than the pooled correlation of comparable previous studies. Hence, besides cell density, other factors, such as necrosis and edema might influence ADC values in glioblastomas.

## Introduction

Glioblastoma (glioma of the WHO grade IV) is the most frequent intrinsic brain tumor with a median overall survival from 12 to 14 months [1, 2]. Only small improvements in overall survival could be achieved by applying aggressive therapy protocols of surgical resection followed by combined radiochemotherapy according to the scheme of Stupp et al [2]. Usually, surgeons strive for complete resection of the tumor, often determined as absence of residual contrast enhancement on intraoperative T1-weighted MRI [3]. But as recurrence often occurs just around the resection cavity in the non-enhancing area, other functional MRI parameters as diffusion-weighted imaging (DWI) might be more suitable to provide preoperative information on the extent of the tumor. DWI is an MR imaging technique that measures the diffusion rate of unbound extracellular water molecules. The DWI-derived apparent diffusion coefficient (ADC) as measure of diffusion of water molecules within each voxel can early-post-surgically be used to assess ischemic areas. Furthermore, it has been hypothesized that the ADC is a useful marker for tumor malignancy and might therefore indicate sites of residual or recurrent tumor in late-post-surgical imaging. The underlying idea is that hypercellularity (increased cell density corresponding to proliferative tumor parts) leads to diminished diffusivity of water by restricting the water diffusivity in the extracellular matrix. Indeed, there are studies that have shown an inverse correlation between the apparent diffusion coefficient (ADC) and tumor cellularity in gliomas [4–17]. Accordingly, ADC has shown to be a promising parameter for predicting outcome in astrocytomas [18–22]. Studies have examined this by calculating the minimum ADC (minADC) of the tumor, the 10^th^ percentile from histogram analysis or determining a cutoff under which overall survival was significantly worse. However, some uncertainty remains in regard to an association of low ADC values with hypercellularity, as there are also studies suggesting that the correlation is positive or non-existent [23–25]. Additionally, it is not fully understood which tissue characteristics play a role in influencing the ADC. Basically, any modification of the extracellular matrix can change the ADC, including extracellular fluid due to vasogenic edema, tumor destruction of the extracellular matrix and necrosis.

In the current study, we applied a largely objective approach to solve this question by automating as many steps of the analysis as possible. Furthermore, we included a total of 561 glioblastoma specimens, exceeding comparable studies by far (Stadlbauer et al. analyzed 77 samples [25]).

## Materials and Methods

### Patients

This retrospective study was approved by the local ethics committee of the University of Heidelberg, Germany. Due to the retrospective nature of the study and the reduced life expectancy of the glioblastoma patients, informed consent was waived. All data were analyzed anonymously. We screened our database of the years 2010–2013 for patients with enhancing brain lesions, suspicious for glioblastoma, that had undergone brain biopsy.for histopathological confirmation. Inclusion criteria were the following: 1) preoperative MR imaging according to the advanced tumor protocol of the neuroradiological department of the University of Heidelberg, including contrast-enhanced T1-weighted imaging and DWI 2) satisfactory quality of the MR images 3) biopsy of the treatment-naïve tumor (for histopathological confirmation of the radiologically suspected glioblastoma) and 4) subsequently histologically confirmed, newly diagnosed glioblastoma.

### MRI scans and ADC maps

All patients underwent preoperative 3 Tesla MRI according to the standardized tumor protocol at our institution (Trio or Verio, Siemens AG Healthcare, Erlangen, Germany)[26]. A 12-channel head matrix coil was used. Among other sequences, ADC-maps based on diffusion-weighted imaging (DWI) (TE = 90ms, TR = 5300ms, voxel size = 1.77mm * 1.77mm * 5.26mm, flip angle = 90°, FOV = (229mm)^2^, matrix size = 130*130, b = 0 and b = 1200 with isotropic gradients) and contrast-enhanced T1-weighted images (cT1) were obtained (TE = 4.04ms, TR = 1710ms, inversion time = 1100ms, voxel size = 0.5mm * 0.5mm * 1.3mm, flip angle = 15°, FOV = (256mm)^2^, matrix size = 512 * 512). All ADC maps were generated on a Siemens Leonardo Station (Siemens AG Healthcare, Erlangen, Germany).

Prior to biopsy, a stereotactic reference frame was mounted on the patient’s head under general anesthesia and intraoperative MRI contrast-enhanced T1-weighted scans (TE = 2.38ms, TR = 9ms, flip angle = 10°, voxel size = (1.035mm)^3^, FOV = (260mm)^2^, matrix size = 256 * 256) were acquired on a 1.5 Tesla MRI scanner (Syngo MR B15, Siemens AG Healthcare, Erlangen) for trajectory planning.

### Stereotactic Biopsies

**Fig 1:**
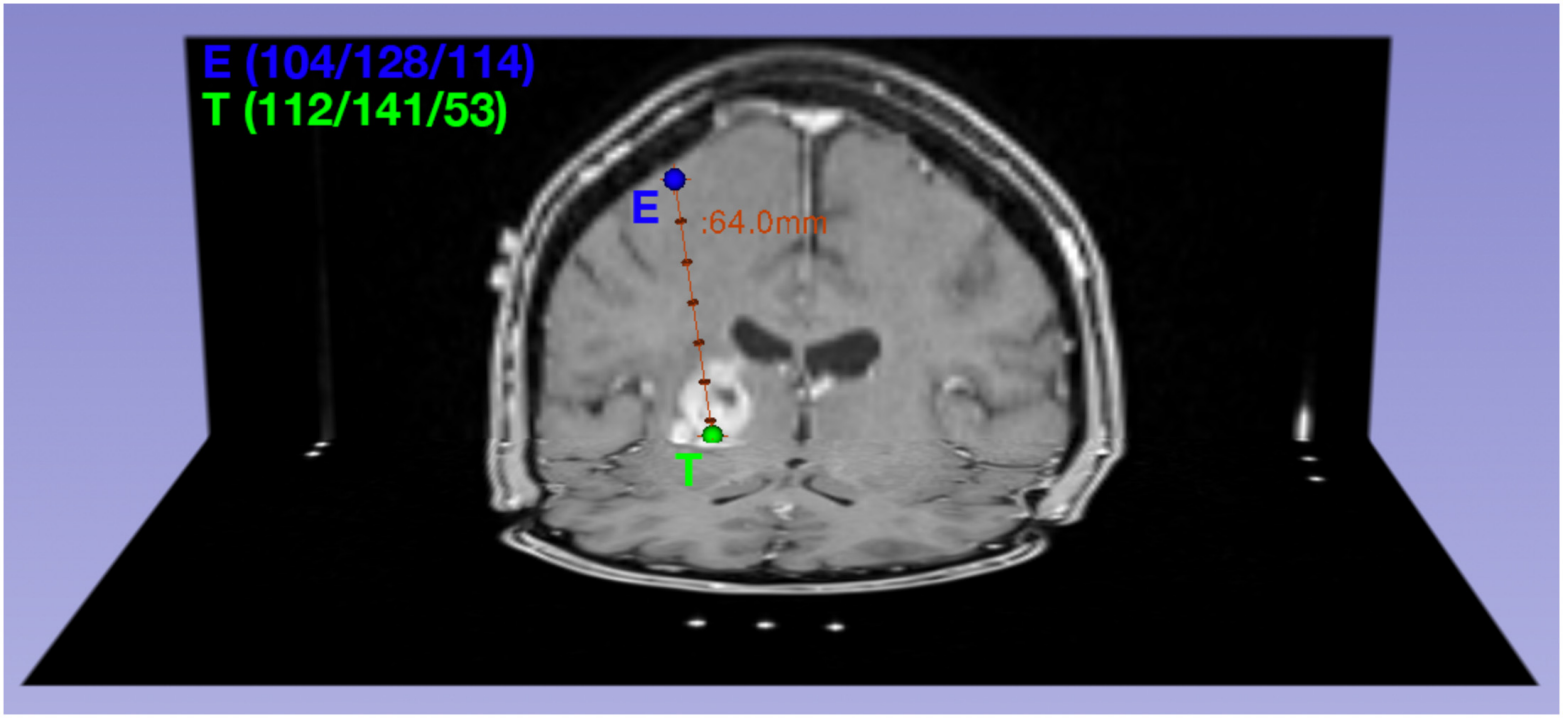
Visualization of a biopsy trajectory. The trajectory is defined by the coordinates of the entry point E(104/128/114) and target point T(112/141/53) and biopsy specimens are taken along the trajectory, mainly close to the target point.

Patients underwent stereotactic biopsy surgery in the Department of Neurosurgery prior to any therapy (Fig 1). Multiple biopsy specimens were taken per patient resulting in a total count of 561 samples that were correlated with their respective preoperative ADC values.

After induction of general anaesthesia, the stereotactic ring was mounted to the head of the patient and intraoperative MRI scans were acquired. Trajectories were planned by the operating Neurosurgeon by setting an entry and target point using the iPS Software (inomed Medizintechnik GmbH, Emmendingen, Germany) which calculated the correct assembly of the stereotactic system for the respective trajectory.

The stereotactic arm (ZD-System, inomed Medizintechnik GmbH, Emmendingen, Germany) was assembled accordingly and the trajectory setting was validated on a target simulator. Following burr hole trephination 9–22 (median: 15) biopsies were taken along the trajectory, ending at the target point in the T1 contrast-enhancing area. Biopsies were labeled so their exact distance to the biopsy target point was known. Biopsy specimens were analyzed at the department of neuropathology. Each biopsy, (approximately (1mm)^3^ in size) was cut in 4–8 slices and stained with hematoxylin and eosin (HE stain). Biopsies were then analyzed and graded as glioblastoma (WHO grade IV) by a neuropathologist. For further post-processing, they were scanned at x20 magnification and saved as NDPI files.

**Fig 2:**
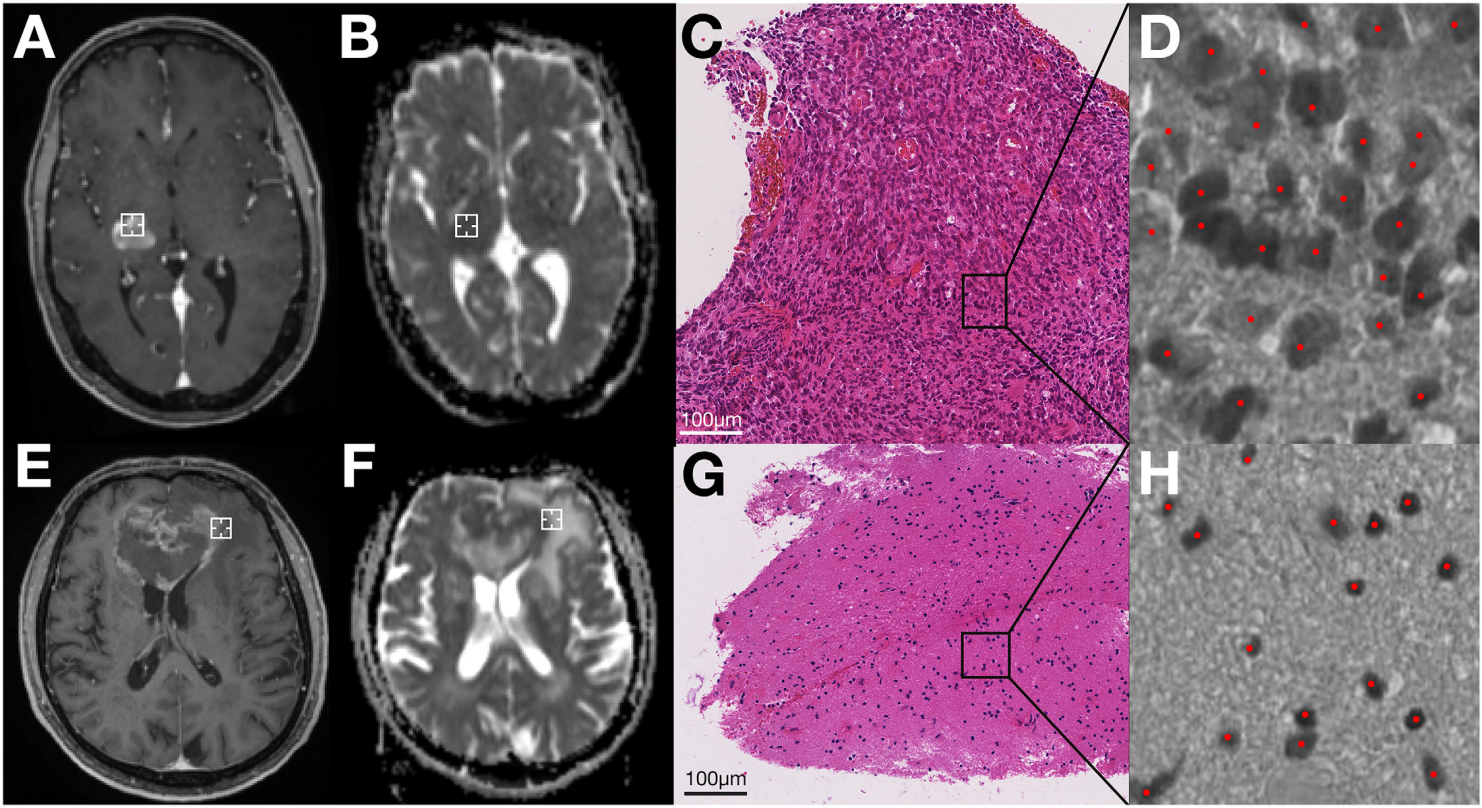
Comparison of two exemplary patients (patient 1: A-D, patient 2: E-H). A,E: Intraoperative cT1-scans. The biopsy location on this slice is marked by a white crosshair. B,F: Preoperative ADC-maps, which have been registered to intraoperative scans as described. The biopsy location on this slice is marked by a white crosshair. C,G: Scanned biopsy specimens of the respective location (HE stain, x20 magnification). D,H: semi-automatic cell counting on 8-bit images by the ImageJ plugin ITCN. Detected cells are marked with red dots. For patient 1(A-D), analysis yielded ADC = 658mm^2^/s and cellularity = 16840 cells/mm^2^. For patient 2 (E-H), it was ADC = 1479mm^2^/s and cellularity = 2208 cells/mm^2^.

Post-processing of the scanned biopsy images was done using NIH ImageJ, 64-bit version [27]. Images were opened using NDPITools [28] or split into smaller-sized images if opening failed. The image was converted to 8-bit and cell density was calculated semi-automatically with the ImageJ plugin ITCN [29], which required an estimate of cell width and cell spacing as input (Fig 2). A neuropathologist who was blinded to the ADC values verified the correctness of cell detection. Cell density was then calculated for each slice per biopsy specimen and averaged.

### Trajectory Analysis

Due to the trajectory being planned based on intraoperative cT1 MRI scans, preoperative scans including the ADC maps had to be coregistered to the intraoperative scans. Therefore, firstly, preoperative ADC maps were aligned and resampled to preoperative cT1 MRI scans. Then, preoperative cT1 MRI scans were coregistered to intraoperative cT1 MRI scans using an affine registration (12 degrees of freedom) in FSL [30]. The computed transformation matrix was saved and also applied to preoperative ADC maps.

ADC value extraction was performed with Matlab (Version 2014b, The MathWorks, Inc., Natick, Massachusetts, USA). The biopsy trajectory from intraoperative MRI cT1 scans with the coordinates of entry point and target point was applied to coregistered preoperative ADC maps using a custom in-house software written in Matlab. ADC values along the trajectory could thus be extracted in 1mm spacing. To account for spatial inaccuracies, the surrounding 26 voxels (3^3^ = 27) of the calculated (1.035mm)^3^ voxel were read out. Thus, the mean ADC value was calculated within a volume of (3.1mm)^3^. This value was then correlated with the cell density of the histological specimen of the respective location which could be reconstructed by vector analysis as the distance between the target point and the biopsy location was given in the pathology report.

### Statistical Analysis

Statistical analysis was performed in R, version 3.2.2. Firstly, the mean ADC and cellularity value per patient was calculated, yielding one pair of values per patient. Based on these 37 pairs, Spearman and Pearson correlation coefficients were calculated and tested. This approach was chosen to compare our results with previous studies. Secondly, the Spearman and Pearson correlation coefficients for all trajectory value pairs (561 in total) were calculated. Thirdly, we applied a linear-mixed-model to the trajectory data including the mean ADC value per patient and the trajectory point specific ADC value differences from the patient mean as predictors as well as a random intercept per patient. For all analyses, we tested the hypothesis that there was a negative relationship between ADC values and cellularity and corresponding one-tailed p values are reported. To account for potential violations of normality, analyses were supplemented with their non-parametric equivalent (Spearman rank correlation) whenever possible.

## Results

Out of 102 patients who had undergone brain biopsy for suspicious glioblastoma in the years 2010–2013, a total of 37 patients (18 male, 19 female, median age 63 ± 13 years) fulfilled the inclusion criteria. On the patient level, the mean ADC value was 1121.88 ± 336.53 mm/s^2^, mean cellularity was 3308.71 ± 1915.32 cells/mm^2^. Details for the ADC and cellularity values in each included patient are summarized in S1 Table.

**Fig 3:**
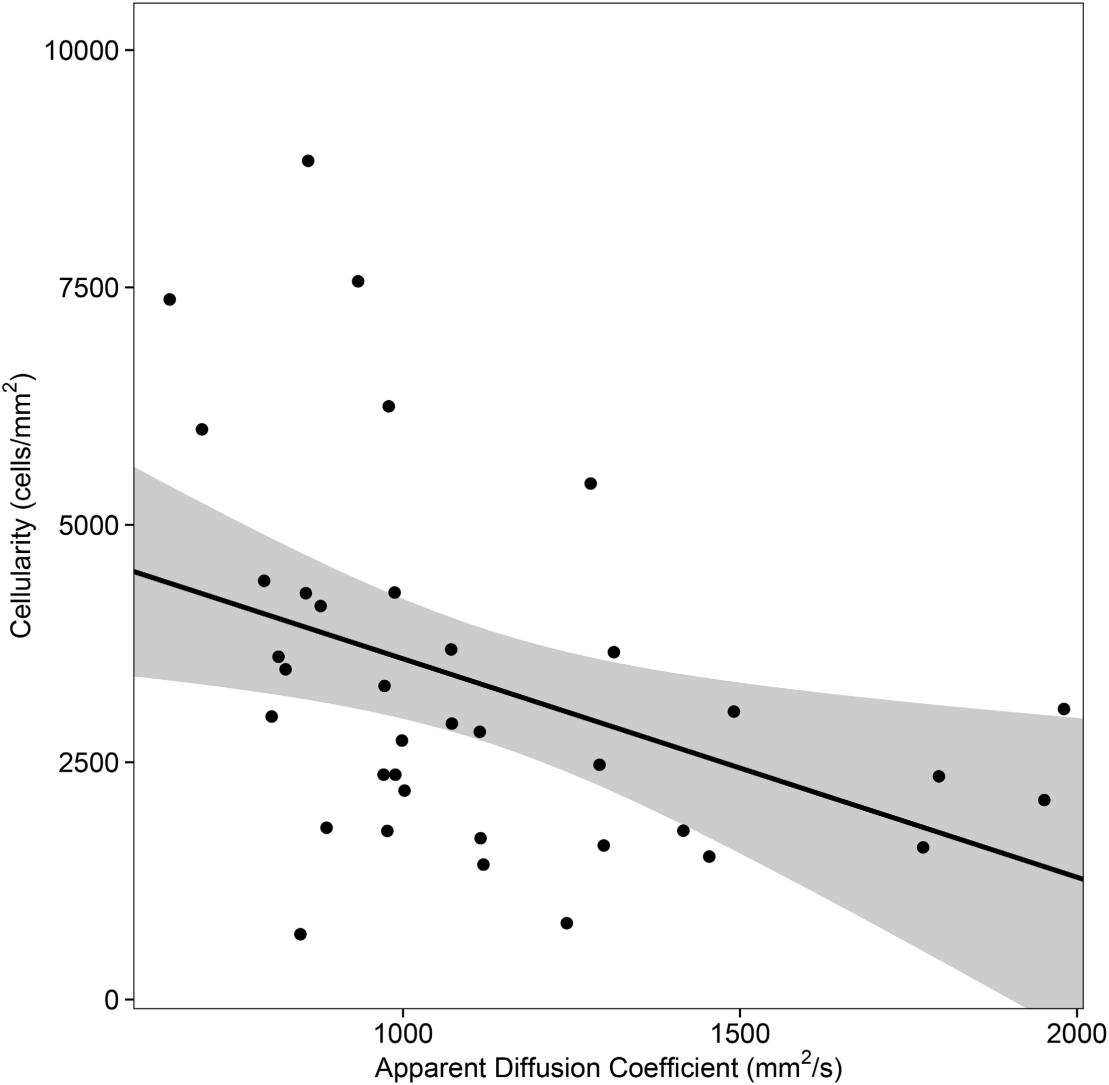
Scatterplot of ADC and cellularity with regression line and 95% confidence interval. Aggregated mean ADC and cellularity values per patient are displayed (37 patients). Pearson’s r = -0.40, p = 0.007; Spearman’s ρ = -0.48, p = 0.002

In the first statistical analysis which included one pair of averaged values per patient, we found a weak to moderate inverse correlation between ADC values and cellularity (Spearman’s ρ = -0.48, p = 0.002; Pearson’s r = -0.40, p = 0.007, Fig 3). Including all trajectory points into a joint analysis and ignoring patient dependencies, the correlation was weaker (Spearman’s ρ = -0.32, Pearson’s r = -0.25, both p<0.001). The linear-mixed-model further supported the previous findings as both the mean ADC value per patient (coefficient estimate = -2.35, p = 0.006) and the trajectory point specific difference from the patient mean (coefficient estimate = -0.89, p = 0.025) significantly negatively predicted cellularity and a stronger negative relationship was observed on the patient level than on the trajectory level.

## Discussion

There is no consensus in literature as to what extent tumor cellularity and ADC values correlate. The hypothesis is that diffusion quantified by the ADC resembles extracellular diffusion. Therefore, ADC values are expected to be low in tumor regions with high cellularity. Reaching a conclusion on this problem would help to validate the clinical value of the ADC. In the current study we found weak inverse correlation between ADC values and cellularity. In the following, we will elaborate how our result complements the research that has been done in that field so far.

Previous studies calculated the correlation by including one pair of ADC and cellularity values per patient. Therefore, we calculated an aggregated mean ADC and cellularity value per patient in our first analysis to be able to compare our results. Furthermore, the correlation was still present (but weaker) when we calculated the correlation of every single ADC-cellularity value pair on the trajectory level. To take patient specific differences into account, we additionally replicated our results in a linear-mixed model where both the mean ADC value and the trajectory point specific differences in ADC were negatively associated with cellularity at the specific trajectory point.

Our aggregated correlation coefficient (ρ = -0.48) is in accordance with the meta-analysis by Chen et al. [4]. However, the pooled correlation coefficient found by Chen et al. is stronger (ρ = -0.61). Possible reasons to account for this discrepancy include our method being less prone to observer-related errors and the optimized spatial congruence of MRI and histology. More precisely, all other studies are at least partially subjected to inter-rater variability due to the applied methods which included that: (a) ADC values were extracted by manually placing a region of interest (ROI) [5–7, 11, 13–17, 24, 31], (b) only one region was analyzed per patient [5–7, 13, 15, 17, 31], (c) high numbers of ADC values in each ROI were averaged (also described as meanADC) [5–7, 11, 13–17, 24, 31] and (d) histological tumor cell counting was performed manually [5–7, 11, 15, 16]. The only study which analyzed ADC and cellularity values in a similar way by registering the trajectory automatically point-by-point and counting cells semi-automatically yielded a positive correlation which was not significant [25]. Additionally, only a few of these studies focused exclusively on glioblastomas. ADC displays different characteristics in different tumor entities, which might bias the correlation analysis; for example, values are significantly lower in lymphomas compared to glioblastomas [13, 32].

One possible explanation for the weak correlation is the tissue heterogeneity within glioblastomas, which includes proliferative cells, extracellular proteins, necrosis in various stadiums and breakdown of the blood-brain-barrier. Besides, it is known that tumor grade is underestimated in 25% of all biopsies which means that features of aggressiveness used for grading might not be present in all areas of the tumor.

There are also conflicting findings regarding ADC values in necrosis. During procedure planning, great care was taken to include both, areas of T1-contrast-enhancement and areas of necrosis, into the biopsy trajectory, because necrosis is a histopathologic feature of glioblastoma. A post-mortem study by LaViolette et al. [33] found low ADC in necrotic regions of glioblastomas while Crawford et al. report significantly elevated ADC values [20], possibly due to degradation of proteins in the extracellular matrix. This could be a possible confounder as cellularity is non-existent or difficult to calculate in necrotic regions and other factors have a bigger influence on the ADC.

A further limitation of our study is the interpolation of ADC values during the coregistration process. Due to the voxel size being larger in ADC maps (1.77mm * 1.77mm * 5.26mm) compared to preoperative cT1 (0.5mm * 0.5mm * 1.3mm) and intraoperative cT1 (1.035mm^3^), the coregistration to the latter led to an interpolation of ADC values across the cT1 image. Furthermore, not all biopsy locations lay exactly on the original MRI slice, so that those ADC values in between slices are once more interpolated values. Additionally, it has to be acknowledged that the ADC voxel volume is larger than the distance between two biopsies. Taking the ADC voxel size (1.77mm * 1.77mm * 5.26mm) and stereotactic biopsy spacing of 1mm into account, the spacing between ADC voxels may be 1.77 to to 5.26 times larger than between biopsies, according to the direction of the trajectory. The selected ADC values may therefore be partially based on interpolation, which constitutes a source of bias.

A brain shift during the biopsy might constitute a further potential bias. However, a relevant brain shift during stereotactic biopsies does usually not occur since the time of dural opening is minimized at the department of neurosurgery at the University of Heidelberg by performing all necessary steps (assembly of targeting device, validation) prior to incising the dura. If relevant CSF leakage is observed, the opening site is immediately sealed with fibrin glue and collagen sponge. Hence, we do not think that brain shift constitutes a significant confounder in this study.

Finally, ADC-values are known to be influenced by many factors apart from necrosis and cellularity such as nuclear-to-cytoplasmic ratio and intra- and extracellular edema [13, 16, 17]. Hence, ADC has to be interpreted as a multifactorial parameter. Additionally, studies have shown that other physiologic MRI parameters emerge as markers for tumor cellularity: Barajas et al. found that rCBV (relative cerebral blood volume) correlates with cell density in the CE part of cT1 [34]. Interestingly, they claim the frequently observed inverse correlation of ADC and cellularity only for the zone of non-contrast enhancement in cT1. Bearing that in mind, the strength of ADC to predict areas of malignancy and infiltration might lie in the combination with other functional MRI parameters, for example from perfusion-weighted imaging, an approach that Barajas et al have pursued [34].

In summary, we provided an objective, observer-independent correlation analysis of ADC and cellularity in a large collective of glioblastoma patients with multiple biopsies. We found an inverse correlation (Spearman’s ρ = -0.48) which is weaker than the pooled correlation coefficient of comparable previous studies. We conclude that the confounding variables on the ADC are various and cannot exclusively be attributed to hypercellularity.

## Supporting Information

S1 Table Minimum, maximum, mean ADC and cellularity values per patient. (DOCX)
