# Supplementary material for: Automatic Analysis of Cellularity in Glioblastoma and Correlation with ADC Using Trajectory Analysis and Automatic Nuclei Counting
Source: PLoS One. 2016 Jul 28;11(7):e0160250. doi: 10.1371/journal.pone.0160250 (PMC4965093; doi:10.1371/journal.pone.0160250)
Supplement: S1 Table — (DOCX) [file pone.0160250.s001.docx]

| **Patient no.** | **Biopsy Count** | | **Min ADC** | **Mean ADC** | **Max ADC** | **Min Cellularity** | **Mean Cellularity** | **Max Cellularity** |
| --- | --- | --- | --- | --- | --- | --- | --- | --- |
|  |  | **mm/s^2^** | | | | **cells/mm^2^** | | |
| 1 | 17 | | 796 | 1278.49 | 1699 | 1518.58 | 5435.67 | 16852.71 |
| 2 | 16 | | 692 | 1071.52 | 1536 | 1936.38 | 3688.21 | 6840.60 |
| 3 | 19 | | 926 | 1072.36 | 1572 | 0 | 2907.66 | 6349.13 |
| 4 | 16 | | 769 | 933.30 | 1042 | 0 | 7564.98 | 11612.66 |
| 5 | 15 | | 892 | 978.75 | 1178 | 0 | 6248.44 | 14762.58 |
| 6 | 9 | | 756 | 847.89 | 942 | 0 | 687.58 | 2047.84 |
| 7 | 17 | | 865 | 1297.91 | 1894 | 0 | 1623.53 | 6993.78 |
| 8 | 14 | | 902 | 971.15 | 1040 | 0 | 2367.93 | 5381.68 |
| 9 | 12 | | 931 | 987.46 | 1195 | 0 | 4287.50 | 8191.71 |
| 10 | 16 | | 874 | 988.58 | 1186 | 1310.34 | 2368.87 | 3741.45 |
| 11 | 17 | | 1226 | 1415.87 | 1539 | 661.46 | 1778.21 | 6833.49 |
| 12 | 12 | | 793 | 886.62 | 1020 | 1069.18 | 1809.85 | 3293.04 |
| 13 | 18 | | 813 | 877.98 | 916 | 0 | 4144.96 | 10732.73 |
| 14 | 16 | | 1129 | 1454.38 | 1623 | 0 | 1506.71 | 2704.45 |
| 15 | 20 | | 680 | 1243.15 | 1551 | 4.93 | 805.02 | 3691.81 |
| 16 | 15 | | 703 | 1002.27 | 1582 | 463.37 | 2201.31 | 5928.76 |
| 17 | 12 | | 1140 | 1951.48 | 2677 | 0 | 2100.23 | 3968.52 |
| 18 | 12 | | 913 | 1980.64 | 2728 | 38.62 | 3059.33 | 7099.23 |
| 19 | 14 | | 658 | 859.37 | 1051 | 0 | 8832.70 | 17256.24 |
| 20 | 18 | | 647 | 805.05 | 1331 | 0 | 2980.19 | 7290.99 |
| 21 | 13 | | 834 | 972.66 | 1119 | 754.62 | 3303.18 | 7008.04 |
| 22 | 12 | | 1675 | 1795.07 | 1973 | 0 | 2350.69 | 3830.47 |
| 23 | 16 | | 619 | 825.83 | 1064 | 27.98 | 3477.87 | 11386.57 |
| 24 | 16 | | 909 | 1115.21 | 1407 | 161.14 | 1700.58 | 3400.89 |
| 25 | 11 | | 651 | 794.10 | 1030 | 467.95 | 4410.16 | 7930.98 |
| 26 | 19 | | 626 | 653.80 | 692 | 2801.17 | 7372.88 | 9563.63 |
| 27 | 14 | | 645 | 701.86 | 800 | 2008.40 | 6005.97 | 9703.74 |
| 28 | 14 | | 948 | 1119.30 | 1354 | 0 | 1421.71 | 3612.35 |
| 29 | 21 | | 1009 | 1490.94 | 2577 | 247.20 | 3032.48 | 7963.87 |
| 30 | 20 | | 783 | 1113.84 | 1499 | 0 | 2819.62 | 7111.31 |
| 31 | 10 | | 828 | 976.71 | 1128 | 179.84 | 1775.40 | 2787.69 |
| 32 | 15 | | 875 | 998.49 | 1103 | 0 | 2728.14 | 4901.75 |
| 33 | 12 | | 1139 | 1291.57 | 1665 | 336.02 | 2472.11 | 3800.25 |
| 34 | 22 | | 785 | 815.28 | 859 | 1755.76 | 3610.88 | 5671.56 |
| 35 | 10 | | 765 | 855.89 | 937 | 1826.82 | 4279.15 | 5667.70 |
| 36 | 20 | | 730 | 1312.86 | 1743 | 0 | 3658.85 | 9640.53 |
| 37 | 11 | | 1332 | 1771.79 | 2129 | 0 | 1603.77 | 10782.56 |
